# Effect of pretreatments of camellia seeds on the quality, phenolic profile, and antioxidant capacity of camellia oil

**DOI:** 10.3389/fnut.2022.1023711

**Published:** 2022-10-12

**Authors:** Mei Wang, Yuancong Zhang, Yin Wan, Qi Zou, Lecheng Shen, Guiming Fu, Er Sheng Gong

**Affiliations:** ^1^State Key Laboratory of Food Science and Technology, College of Food Science and Technology, Nanchang University, Nanchang, China; ^2^International Institute of Food Innovation, Nanchang University, Nanchang, China; ^3^State Center of Quality Testing and Inspection for Camellia Products, Ganzhou, China; ^4^Ganzhou General Inspection and Testing Institute, Ganzhou, China; ^5^School of Public Health and Health Management, Gannan Medical University, Ganzhou, China

**Keywords:** camellia oil, puffing, oil yield, phenolics, sterols, antioxidant capacity

## Abstract

Camellia oil is one of the four major woody oils in the world and has high nutritional value due to its richness in monounsaturated fatty acids (MUFAs) and bioactive substances. In order to compare the effects of pretreatments of camellia seeds on the quality, phenolic profile, and antioxidant capacity of camellia oil, three different pretreatment methods, i.e., hot air (HA), steam (ST) and puffing (PU), were used to treat the seed powder in the present study. All three pretreatments changed the internal structure of the camellia seeds. The oil yield was increased after all three pretreatments, with the highest oil yield increased by PU pretreatment (Based on the oil yield, we screened out the best conditions of the three pretreatments, HA pretreatment is 60°C for 40 min, ST pretreatment is 100°C for 15 min, PU pretreatment is 800 rpm). The fatty acids (FAs) of the oil were relatively stable, with no significant changes after three pretreatments. However, all three pretreatments had a significant effect on the acid value (AV), peroxide value (PV), and benzo(a)pyrene (Ba P) of the camellia oil. The PU and HA pretreatments could increase the tocopherol content and the total sterols content in the camellia oil. The ST and PU pretreatments significantly increased the free phenolics (FP) content, all three pretreatments reduced the contents of conjugated phenolics (CP) and insoluble-bound phenolics (IBP) in the camellia oil. The IBP made the most significant contribution to the antioxidant capacities of camellia oil. ST and PU prtreatments increased the antioxidant capacities of the total phenolics in the camellia oil. Eight phenolics in FP, CP, and IBP were significantly correlated with the antioxidant capacities of camellia oil. The results of the present study could provide some theoretical guidance for the pretreatment of camellia seeds for higher oil yield, phenolic content and enhanced antioxidant capacities of camellia oil.

## Introduction

Camellia oil (*Camellia oleifera Abel*.) is one of the four major woody seeds oil, its oil content is about 30% (1). It is considered as a high-grade edible oil due to its suitable fatty acid (FA) composition and richness in a variety of bioactive substances. These components with various biological activities are useful for lowering blood pressure and cholesterol, protecting the liver, fighting against cancer, and alleviating gastrointestinal pain (2). In addition to its genetic properties, the physiological activity of camellia oil is also influenced by the oil production process (3) and pretreatment methods (4). The traditional and popular method of making oil is cold pressing. Cold pressed camellia oil is thought to have high content of bioactive compounds as it is produced without heat treatment. However, recent studies have shown that high temperature pretreatment of the seeds prior to oil extraction could significantly increase the amount of bioactive compounds in oil (5).

In the production of edible oils, the pretreatment process is an important step and has a significant impact on the yield and quality of oil (6). At present, the pretreatments of camellia seeds includes roasting, hot air drying, steam treatment, microwave treatment, infrared treatment, steam explosion and so on. Many researchers have found that different pretreatments have different effects on the quality of oil. Ji et al. (7) found that the contents of tocopherols and sesamin in sesame oil were decreased with increasing temperature of heating, but the formation of sesaminols in sesame oil could be promoted when the temperature was increased to a certain level. Yang et al. (8) found that the roasting temperature influenced the total phenolic content (TPC) and total flavonoid content of camellia oil, but the roasting temperature did not influence the FA profile and tocopherol content. Zhang et al. (9) found that steam explosion pretreatment not only increased the oil yield of the camellia seeds, but also increased the physicochemical properties and antioxidant activities of camellia oil.

Phenolics, a kind of important antioxidant components in camellia oil, have the functions of scavenging free radicals, delaying oil self-oxidation, and inhibiting the formation of oxidation products (10). According to whether they are bound to other substances in the plant matrix, phenolics are classified into free phenolics (FP) and bound phenolics (BP) (11). Some soluble phenolics are esterified to sugars, which are defined as conjugated phenolics (CP). There are three main forms of phenolics presented in camellia seeds, namely FP, CP and insoluble-bound phenolics (IBP) (12). Pretreatment has a positive effect on the oil, but previous studies have concentrated more on a single method of pretreatment, and rarely compared different pretreatment methods. The effect of pretreatment on the profiles and antioxidant capacities of phenolics in camellia oil are also less studied. In the present study, we used three different pretreatment methods, including two traditional pretreatment methods, hot air (HA) and steam (ST), and a new pretreatment method, puffing (PU), to treat the camellia seed powder, to investigate the effect of pretreatments of camellia seeds on the quality, and phenolic profile, and antioxidant capacity of camellia oil.

## Materials and methods

### Chemicals and reagents

Methanol, n-hexane, and ethyl acetate were purchased from Thermo Fisher Scientific Co. Ltd. (Massachusetts, USA). Petroleum ether, hydrochloric acid, sodium hydroxide, sodium carbonate, Folin–Ciocalteu reagent, sodium acetate, acetic acid, and ferric chloride were purchased from Beijing Sinopharm Chemical Reagent Co. Ltd. (Beijing, China). TPTZ (2,4,6-Tripyridyltriazine), Trolox (6-Hydroxy-2,5,7,8-tetramethylchromane-2-carboxylic acid), DPPH (2,2-Diphenyl-1-picrylhydrazyl), ABTS (2,2′-Azobis(2-methylpropionamidine) dihydrochloride) and potassium persulfate were purchased from Shanghai Macklin Biochemical Co. Ltd. (Shanghai, China).

### Camellia seeds samples preparation

Camellia fruits were harvested from the camellia base of Jiangxi Xingguo Jiaxiangle Food Company (Ganzhou, China) in October 2019. The harvested camellia fruits were dried at 60°C in a drying machine (Anlu, China) and then peeled to obtain camellia seeds, whose final moisture content was 7–9% (in dry basis). The dried camellia seeds were stored in the brown glass bottles at 4°C for further use.

### Pretreatment of camellia seeds and oil extraction

After crushing the camellia seeds with a crusher, HA, PU, ST were used to pretreat the camellia seed powder. The pretreated and untreated camellia seeds powder were pressed to make oil using a hydraulic oil press (6YY-150, Henan Province Gongyi City Luyuan Machinery Company, Gongyi, China). The camellia oil obtained at the optimal condition of each pretreatment was used for further analysis.

#### HA pretreatment of camellia seeds

The camellia seeds powder was treated with hot air in a drying oven (DHG-9140A, Shanghai Sanfa Scientific Instruments Company, Shanghai, China) at 60, 100, 120, and 150°C for 40 min.

#### PU pretreatment of camellia seeds

The camellia seeds powder was puffed by an extruder (MKJ-100, Anlu Tianxing Grain and Oil Machinery Equipment Company, Anlu, China) at 600, 800, 1,000, 1,200, and 1,400 rpm.

#### ST pretreatment of camellia seeds

The camellia seeds powder was steamed with an electric steamer (ST-47, Chaozhou Chaoan District Sutai Hardware and Electrical Appliance Factory, Chaozhou, China) at 100°C for 5, 10, 15, 20, and 30 min.

### Determination of oil yield of camellia seeds

Camellia seeds powder and cake after oil extraction were collected. The oil contents of camellia seeds and cakes were determined with reference to ISO 659-2009, the oil was extracted from a test portion in a suitable apparatus with hexane or light petroleum, and the solvent was removed from the extract and the extract was weighed. The moisture content of camellia seeds was determined with reference to ISO 665-2000, the seeds was dried at 103 ± 2°C in an oven at atmospheric pressure, until practically constant mass was reached. The oil content of camellia seeds and cakes were calculated on a dry basis with the water removed according to the following formula:


$$\begin{array}{l} \text{oil content\% }\left(\text{in dry basis}\right)=\frac{\text{weight of the oil collected}}{\text{weight of camellia seeds}}\times 100\%. \end{array}$$

The oil yield of camellia seeds was the oil content of camellia seed minus the oil content of camellia cake.

### Determination of physicochemical properties, FA composition, and bioactive compounds of camellia oil

Acid value (AV) was determined with reference to ISO 660-2020, the sample was dissolved in a solvent mixture of ethanol and diethyl ether, and the acids are titrated with an ethanolic or methanolic solution of potassium or sodium hydroxide. Peroxide value (PV) was determined with reference to ISO 3960-2017, the sample was dissolved in isooctane and glacial acetic acid, and potassium iodide was added, the iodine liberated by the peroxides was determined iodometrically with starch indicator and sodium thiosulfate standard solution, the endpoint of the titration was determined iodometrically. Benzo(a)pyrene (Ba P) content was determined with reference to ISO 15302-2017, the sample was dissolved in light petroleum, a suitable amount of sample was adsorbed on an alumine column and eluted with light petroleum to remove any benzo(b)pyrene. FAs, tocopherols, sterols, and squalene in camellia oil were determined using the methods previously reported by Ye et al. (13).

### Seed microstructure

Camellia seed samples (raw and pretreated) were crushed and freeze dried for 24 h. A scanning electron microscope (SEM, JSM 6701F, JEOL Japan Electronics Co., Ltd., Tokyo Akishima Station, Japan) was used to observe the microstructure of camellia seed at an acceleration potential of 5.0 kV. The camellia seed was placed on a double-sided adhesive tape attached to a metal stub and coated with 20 nm gold under vacuum. The magnification of panoramic and regional images was 100×, 500×, and 1,000×, respectively.

### Extraction and determination of different forms of phenolics in camellia oil

**Figure 1:**
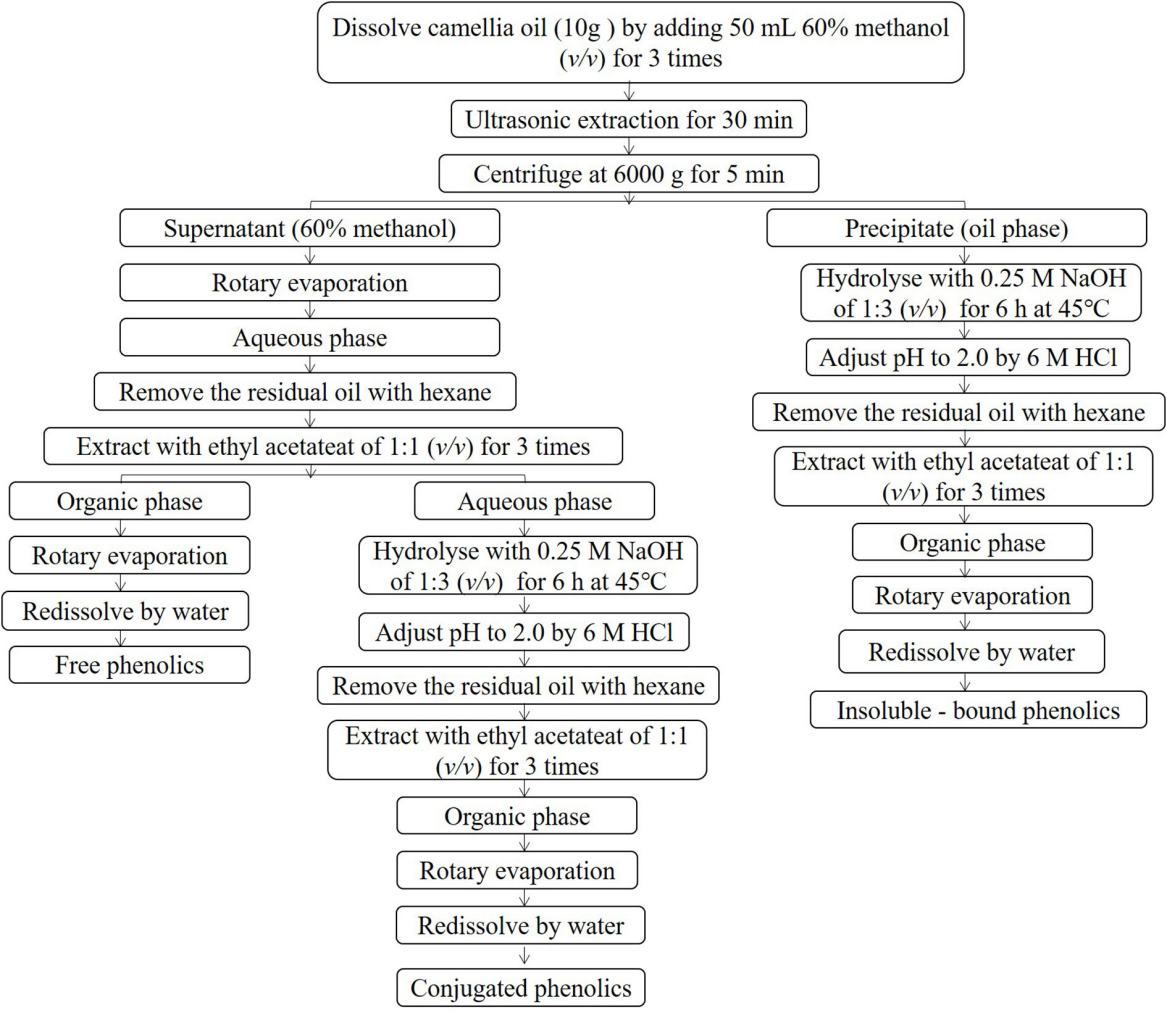
Extraction procedure of the three forms of phenolics in camellia oil.

The extraction of different forms of phenolics from camellia seed oil was carried out according to the methods reported by Kang (14) and Ayoub et al. (15). The extraction procedure for the three forms of phenolics is shown in Figure 1.

#### Extraction of free phenolics

Ten gram of camellia oil sample was blended with 50 mL of 60% methanol solution under ultrasonication for 30 min followed by centrifugation at 6,000 *g* for 5 min. The supernatant was collected and the residue was reextracted for 3 times. The supernatants were combined, and evaporated in a rotary evaporator (Digital G3, Heidolph Instruments GmbH & CO. KG, Schwabach, Germany) at 40°C. Hexane was added to the aqueous phase to remove the residual oil. Ethyl acetate was added to the mixture at a ratio of 1:1 followed by centrifugation at 6,000 *g* for 5 min. The supernatant was collected and the residue was reextracted for 3 times. The supernatants were combined and evaporated at 40°C to dryness, and reconstituted to 5 mL with distilled water.

#### Extraction of conjugated phenolics

The residual aqueous phase after ethyl acetate extraction in the FP extraction was blended with 0.25 mol/L NaOH solution at a ratio of 1:3 at 45°C for 6 h. The mixture was adjusted to pH 2 using hydrochloric acid and then extracted with hexane to remove the residual oil. The aqueous phase was repeatedly extracted for 3 times using ethyl acetate at a ratio of 1:1 followed by centrifugation at 6,000 *g* for 5 min. The supernatants were combined and evaporated at 40°C to dryness, and reconstituted to 5 mL with distilled water.

#### Extraction of insoluble-bound phenolics

The residual oil phase after 60% methanol extraction in the FP extraction was blended with 0.25 mol/L NaOH solution at a ratio of 1:3 at 45°C for 6 h. The mixture was adjusted to pH 2 with hydrochloric acid and then extracted with hexane to remove the residual oil. The aqueous phase was repeatedly extracted for 3 times using ethyl acetate at a ratio of 1:1 followed by centrifugation at 6,000 *g* for 5 min. The supernatants were combined and evaporated at 40°C to dryness, and reconstituted to 5 mL with distilled water.

#### Determination of TPC

TPC of phenolic extract was determined according to the method reported previously (16). One milliliter of sample extracts or standard solutions of gallic acid were reacted with 0.5 mL of Folin-Ciocalteu reagent for 6 min. Two milliliters of 7.5% sodium carbonate solution and 6.5 mL of water were added to the mixture. The blue color of the solution was developed for 90 min, and measured at 760 nm using an UV spectrophotometer (Purkinje General Instrument Co., Ltd., Beijing, China). TPC was expressed as mg of gallic acid equiv (GAE)/100 g oilseed dry weight (DW).

#### Identification and quantification of phenolics

One hundred microliter of phenolic extracts was diluted using 400 μL of methanol. The mixture was placed in an ice bath for 5 min followed by centrifugation at 15,000 *g* for 20 min at 4°C. The supernatants were used for HPLC-MS/MS analysis. LC-MS/MS analysis was performed on an ExionLC™ AD system (SCIEX, Framingham, USA) coupled with a QTRAP^®^ 6500+ mass spectrometer (SCIEX, Framingham, USA). Samples were injected into an Xselect HSS T3 column (2.1 × 150 mm, 2.5 μm) using a 20 min linear gradient at a flow rate of 0.4 mL/min for the positive/negative polarity mode. The eluents were made up of 0.1% formic acid solution in water (A) and 0.1% formic acid solution in acetonitrile (B) (17). The solvent gradient was set as follows: 0–2.0 min, 2% B; 2.0–15.0 min, 2–100% B; 15.0–17.0 min, 100% B; 17.0–17.1 min, 100–2% B; 17.1–20.0 min, 2% B. QTRAP^®^ 6500+ mass spectrometer was operated in positive polarity mode with curtain gas of 35 psi, collision gas of medium, ion spray voltage of 5,500 V, temperature of 550°C, ion source gas of 1:60. QTRAP^®^ 6500+ mass spectrometer was operated in negative polarity mode with curtain gas of 35 psi, collision gas of medium, ion spray voltage of −4,500 V, temperature of 550°C, ion source gas of 1:60.

The detection of phenolics using MRM (Multiple Reaction Monitoring) was based on Novogene database (Novogene Bioinformatics Technology Co. Ltd., Beijing China). The Q3 were used for the quantification of phenolics. The Q1, Q3, RT (retention time), DP (declustering potential) and CE (collision energy) were used for the identification of phenolics. The data files generated by HPLC-MS/MS were processed using the SCIEX OS Version 1.4 to integrate and correct the peak. The main parameters were set as follows: minimum peak height, 500; signal/noise ratio, 5; gaussian smooth width, 1. The area of each peak represents the relative content of the corresponding phenolic.

### Determination of antioxidant activity

#### Determination of DPPH free radical scavenging capacity

DPPH free radical scavenging capacity was determined using the method reported by Brand-Williams et al. (18). Briefly, 3 mL of 60 μmol/L DPPH radical solution in alcohol was mixed with 100 μL of phenolic extract or Trolox standard solutions. The absorbance was measured at 517 nm after 30 min of reaction at room temperature. The DPPH radical scavenging capacity was expressed as μmol of Trolox equivalent (TE)/100 g oil.

#### Determination of ABTS free radical scavenging capacity

ABTS free radical scavenging capacity was determined according to the method reported by Re et al. (19). Briefly, 50 mL of 7 mmol/L ABTS solution was mixed with 880 μL of 2.45 mmol/L potassium persulfate in a homogeneous solution, which was left overnight at 25°C. Then the mixture was mixed with methanol at a ratio of 1:1 to stabilize its absorbance in the range of 0.7 ± 0.05 before use, and this solution was the ABTS radical working solution. The ABTS radical working solution was mixed with 100 μL phenolic extracts or Trolox standard solutions. The absorbance was measured at 734 nm after 30 min at room temperature. The ABTS radical scavenging capacity was expressed as μmol of TE/100 g oil.

#### Determination of ferric reducing antioxidant power

Ferric reducing antioxidant power (FRAP) was determined following the method described by Benzie and Strain (20). Briefly, acetate buffer (300 mmol/L, pH 3.6), 20 mmol/L FeCl_3_ solution, and 10 mmol/L TPTZ solution (in 4 mmol/L HCl) were mixed at a ratio of 10:1:1 to obtain FRAP working solution. Then, 2.4 mL of standard solutions or phenolic extract was mixed with 0.1 mL of FRAP working solution. The mixture was incubated at 37°C for 10 min. The absorbance was measured at 593 nm. FRAP was expressed as μmol of TE/100 g oil.

### Statistical analysis

Results are presented as the mean ± SD from three independent experiments. The data were fitted and plotted using Origin 9.0 (OriginLab, Northampton, MA, USA). Differences among different samples (oils from UT, and HA, ST, PU pretreated seeds) were analyzed by the Tukey's pairwise comparisons of ANOVA with significance level of *P* < 0.05 using SPSS 22.0 (IBM Corp., NY, USA). OPLS-DA analysis was conducted using the Wekemo Bioincloud (www.bioincloud.tech).

## Results and discussion

### Effect of HA, ST and PU pretreatments on oil yield and moisture content of camellia seeds

**Figure 2:**
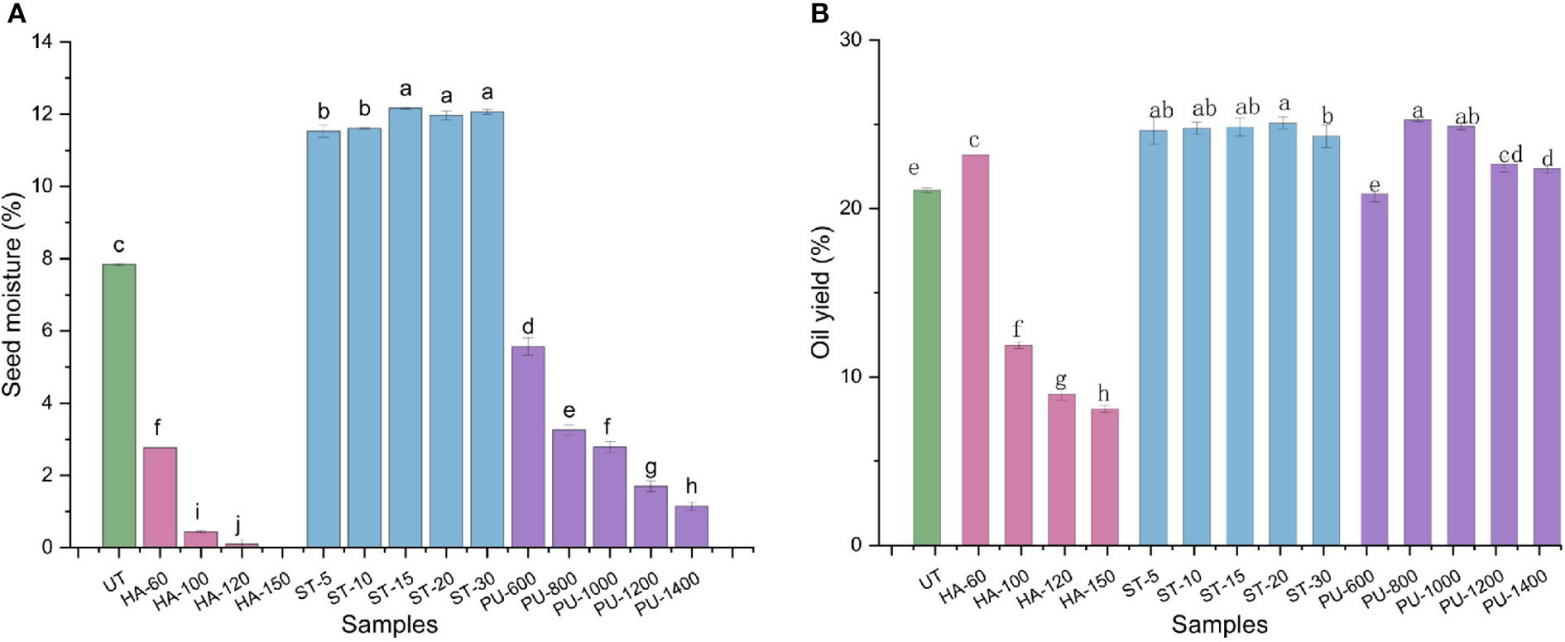
The moisture content **(A)** and oil yield **(B)** of camellia seeds treated with different pretreatment methods (UT, untreated; HA, hot air; PU, puffing; ST, steam). Bars with no letter in common are significant different (*P* < 0.05). The numbers following the abbreviations represent the processing conditions for each pretreatment method.

Oil yield is an important variable to seed oil processors as it is one of the key profit determinants for the business. Moisture content is a factor that affect oil yield during expression processing of oil seeds (6). To investigate the effect of HA, ST and PU pretreatments on the moisture content and oil yield of camellia seeds, pretreated and untreated (UT) camellia seed samples were pressed separately to calculate their oil yield and to find out the optimal conditions for each pretreatment method. The results are shown in Figure 2. The camellia seed powder was treated with HA at four different temperatures (60, 100, 120, and 150°C). With the increase of temperature, the moisture content of camellia seed was decreased sharply, and the oil yield of camellia seed also showed a downward trend, probably because the high temperature caused the loss of water in the camellia seeds, and the change in water would eventually lead to a decrease in oil yield (13). HA pretreatment of 60°C for 40 min for camellia seed powder was chosen as the optimal condition. Compared to UT seeds, the oil yield of HA treated seeds was increased by 9.91%.

ST pretreatment is a kind of hydrothermal pretreatment. The camellia seed powder was treated with five different durations of steam. It was found that the oil yield did not change significantly with time, and the moisture content also changed a little. It's probably because the main purpose of ST pretreatment is to break the cell wall by using temperature, so that the oil can be more easily spilled, and the ST pretreatment after 5 min had already made the cell wall fully ruptured. So the moisture content of the seeds remained unchanged for the subsequent extension of time, and therefore the oil yield was increased. ST 15 min for camellia seed powder was chosen as the best pretreatment condition for ST pretreatment, the oil yield of camellia seed was increased by 17.78% under this condition in comparison with the UT camellia seed.

After PU pretreatment with five different speeds, the oil yield was firstly increased and then decreased, while the moisture content was decreased as the speed was increased. The oil yield of PU pretreated camellia seeds was higher than that of the UT camellia seeds except for PU-600 pretreated camellia seeds, whose oil yield was slightly lower than that of UT camellia seeds (*P* > 0.05). This may be due to that the oil cannot fully overflow from the seeds at low speed (600 rpm), thus resulting in a low oil yield. At 800–1,000 rpm, the oil could fully burst out of the seeds, so the oil yield was higher than UT camellia seeds. The higher the speed instantaneous expansion, the higher the temperature is. When the oil is squeezed to the outlet, as the pressure inside and outside of the oil seed instantly changed from high pressure to atmospheric pressure, the internal water evaporated out rapidly, which might eventually lead to a decrease in oil yield (21). Therefore, the speed of 800 rpm was selected as the best condition for PU pretreatment, and the oil yield of camellia seeds after PU pretreatment was increased by 19.82% in comparison with UT camellia seeds. In general, the oil yields of all three pretreated camellia seeds at optimal condition were increased in comparison with UT camellia seeds, with the greatest increase after PU pretreatment, followed by ST pretreatment and HA pretreatment.

### Effect of pretreatments on FAs of camellia oil

The optimum conditions of camellia seed powder pretreatments for oil yield were selected, namely 60°C HA pretreatment for 40 min, ST pretreatment for 15 min and PU pretreatment at 800 rpm. The camellia oil obtained from these treated camellia seed powder and the untreated camellia seed powder were used for subsequent analyses.

**Figure 3:**
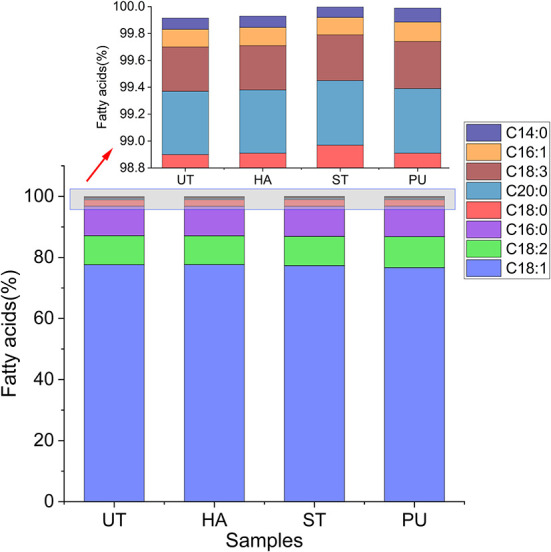
Fatty acid composition of camellia oils from untreated and HA, ST, PU pretreated camellia seeds.

The FA composition of the camellia oil after HA, ST and PU pretreatments is shown in Figure 3. The major FA of camellia oil was oleic acid (76.65–77.68%), followed by palmitic acid (9.68–9.99%) and linoleic acid (9.41–10.17%). The unsaturated FA (UFA, include palmitoleic acid, oleic acid, arachidonic acid, erucic acid, tetracycoenoic acid, linoleic acid and linolenic acid) content exceeded 80% of the total. The saturated FA (SFA, include cardamic acid, palmitic acid, stearic acid and arachidonic acid) content was <20% of the total. The FA content of camellia oil was similar to the results of Ye et al. (13). The FAs of the camellia oil were relatively stable, with no significant changes in oleic acid-based monounsaturated FA (MUFA), linoleic acid-based polyunsaturated FA (PUFA), and palmitic acid-based SFA after HA, ST and PU pretreatments. Similarly, Kraljić et al. (5) and Wroniak et al. (21) also demonstrated no significant changes in FA composition of the oil after roasting and conventional oven heating of seeds.

### Effect of pretreatments on camellia seed microstructure

**Figure 4:**
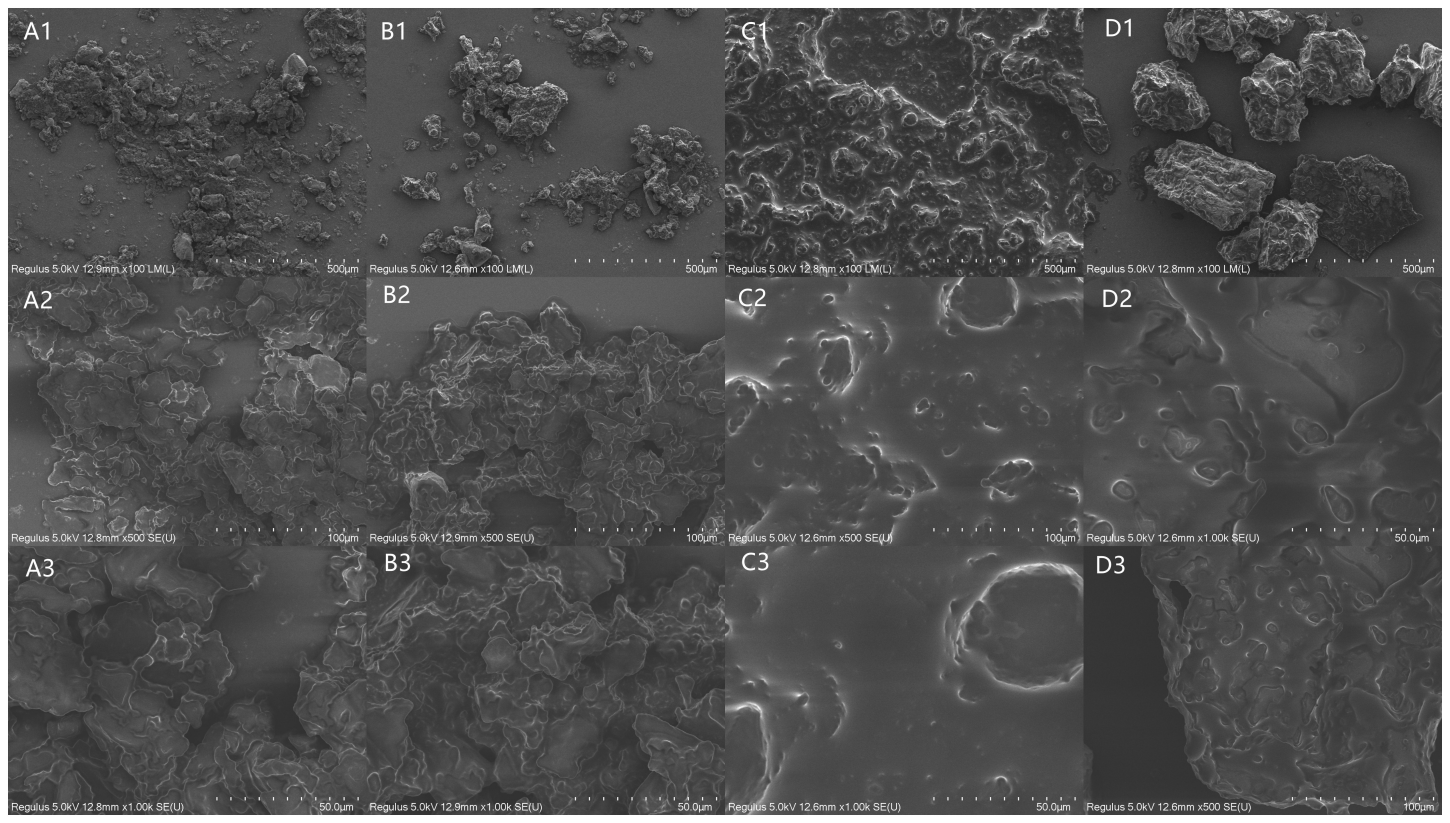
Scanning electron microscopy (SEM) images of untreated and three pretreated camellia seeds. The letters present the treated methods, [**(A)** UT, **(B)** HA treated, **(C)** PU treated, **(D)** ST treated camellia seeds]. The numbers present the magnification, 1 is 100 ×, 2 is 500 ×, 3 is 1,000 ×.

Figure 4 illustrates the SEM images of the untreated and pretreated camellia seeds. The internal structure of the untreated camellia seed was intact with no obvious damage (Figure 4A). The intact cell walls and adherent membranes contribute the main resistance to extrusion for oil production (22). Three pretreatments had different degrees of effects on the internal structure of the seeds. As shown in Figure 4B, HA pretreatment resulted in greater porosity and more irregular pores in the internal structure of the camellia seeds. This may be due to the structural changes in cells caused by thermally induced denaturation and the damages of protein and lipid (13). After PU pretreatment (Figure 4C), there was obvious some oil, which may be due to the transient high rotational speed causing oil to be puffed out of the cells. ST treated seeds also had some oil as the PU treated seeds, but unlike the PU treated seeds, the ST treated seeds had more aggregation and agglomeration (Figures 4D,D1), which may be caused by the higher moisture content in the camellia seed powder.

### Effect of pretreatments on the AV, PV, and Ba P of camellia oil

**Figure 5:**
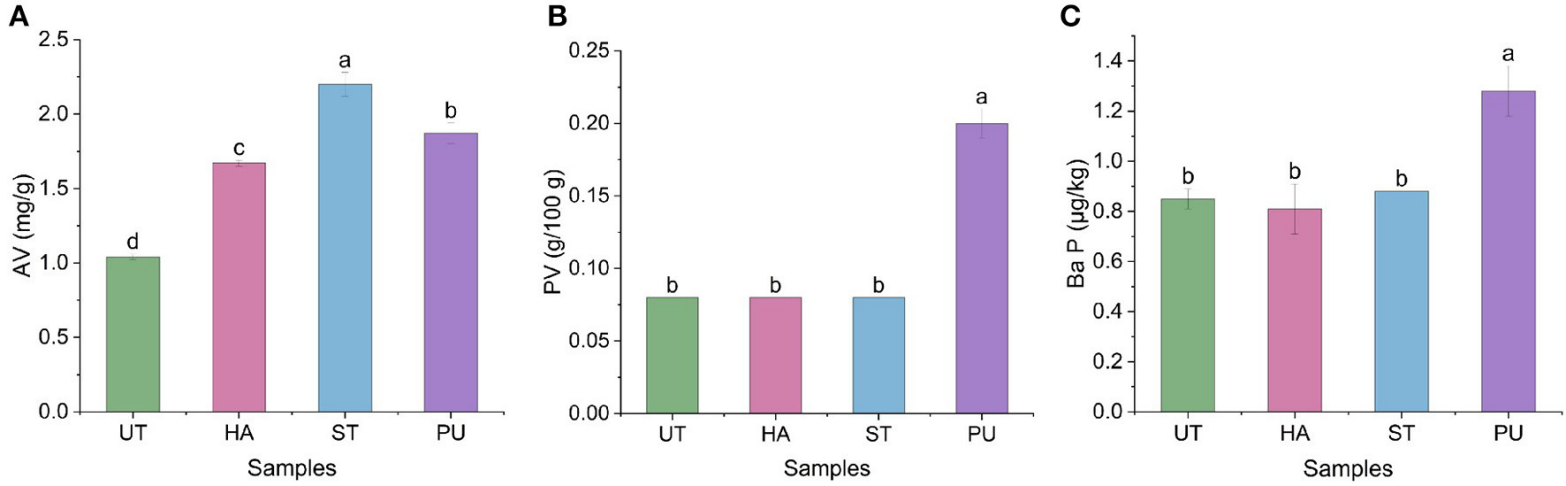
The acid value (AV), peroxide value (PV), and Benzo(a)pyrene (Ba P) of camellia oil from untreated and HA, ST, PU pretreated camellia seeds. [**(A)** AV, **(B)** PV, **(C)** Ba P]. Bars with no letter in common are significant different (*P* < 0.05).

The AV and PV are two important parameters for assessing the quality of edible oils. Ba P is one of the most representative and strong carcinogens among more than 20 carcinogenic polycyclic aromatic hydrocarbons that have been studied (23). These three indicators were measured to evaluate the quality of the oil in the present study and the results are shown in Figure 5.

As shown in Figure 5A, all three pretreatments increased the AV of camellia oil. The AV of ST treated camellia oil was the highest (2.2 mg/g), followed by PU treated and HA treated camellia oil. The increase in the AV of camellia oil might be attributed to hydrolysis of triacylglycerols, which could produce free FAs. According to the results of PV (Figure 5B), HA and ST pretreatments basically did not affect the PV of the camellia oil, while PU pretreatment significantly increased the PV of camellia oil. Uquiche et al. (24) also investigated the effect of pretreatment on hazelnuts and rapeseeds, and found that AVs of hazelnuts and rapeseeds oil was significantly increased, while PVs of hazelnuts and rapeseeds oil was not significantly affected. Similar to the PV results, HA and ST pretreatments basically did not affect the Ba P content of camellia oil, while PU pretreatment significantly increased the Ba P content. This may be due to the instantaneous local overheating during the expansion, which resulted in the pyrolysis of organic matter and the formation of Ba P. In a study on the effect of roasting on Ba P in sesame oil, Cheng et al. (25) found that Ba P content was increased with increasing roasting time and temperature, and reached up to about 5 μg/kg. The Ba P content of camellia oil after PU pretreatment was much lower than their result, probably attributed to the transient high temperature of the PU pretreatment. In summary, the three pretreatments increased the AV of camellia oil, while PU pretreatment increased the PV and Ba P of camellia oil. But both AV and PV of camellia oil were lower than the Chinese national standard GB/T 11765-2018 for AV (≤ 3 mg/g) and PV (≤ 0.25/100 g), and Ba P content of camellia oil after pretreatments were far lower than the Chinese national standard GB 2762-2017 (≤ 10 μg/kg).

### Effect of pretreatments on bioactive compounds of camellia oil

Camellia oil contains many bioactive compounds, including tocopherol, squalene, sterol, and phenolics. The content of bioactive substances is not only related to the property of camellia oil, but also related to the production and processing technology of camellia oil. Recent studies suggested that after thermal pretreatments of seeds *via* oven heating (22) or microwave (13), greater amount of bioactive compounds may penetrate into the produced oil. Therefore, the contents of tocopherols, squalene, and sterols in camellia oil with or without pretreatment were determined to investigate the effect of pretreatments on the contents of bioactive substances of camellia oil.

#### Tocopherols of camellia oil

**Figure 6:**
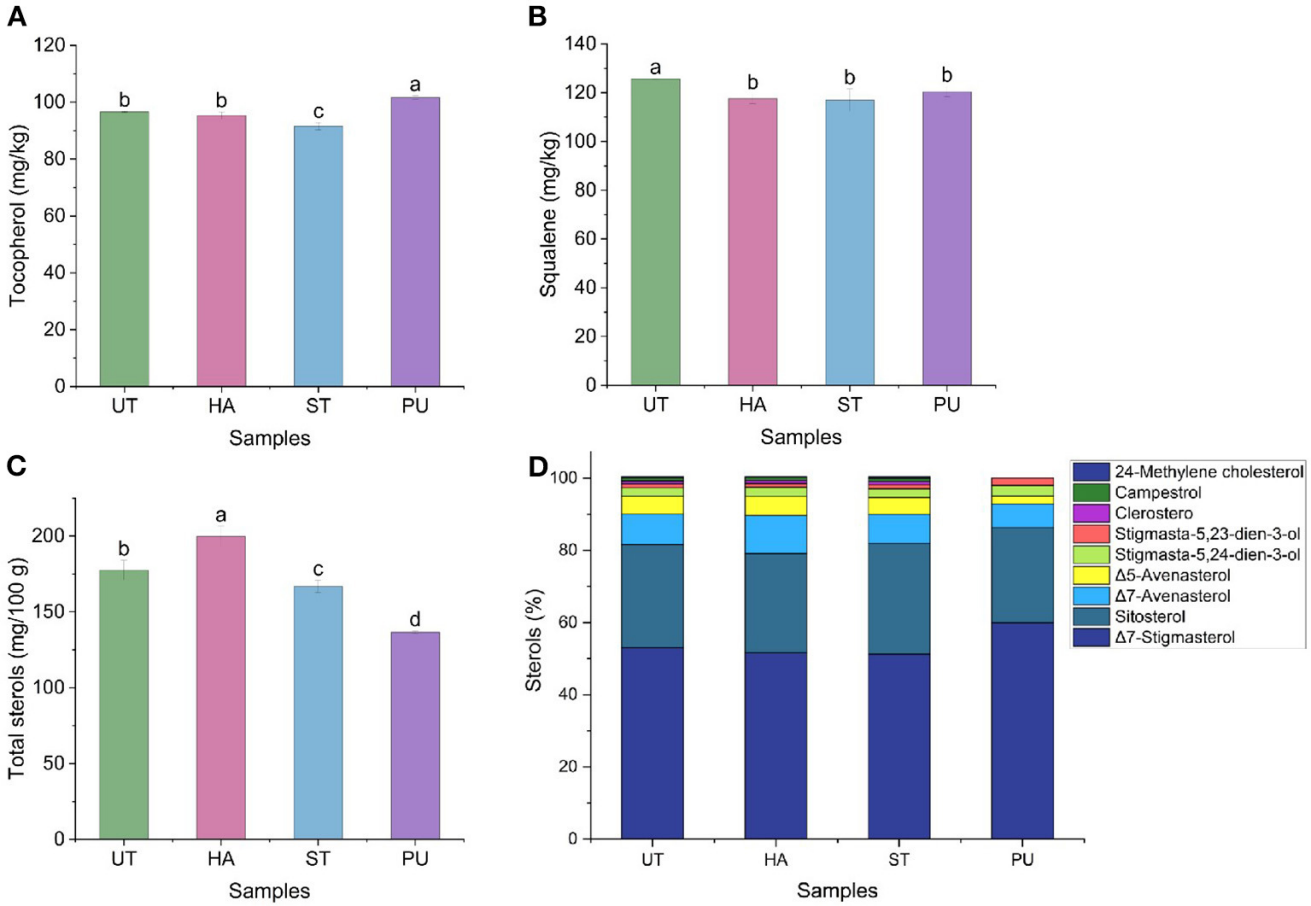
The contents of tocopherols, squalene, and sterols of camellia oil from untreated and HA, ST, PU pretreated camellia seeds [**(A)** tocopherol, **(B)** squalene, **(C)** total sterols, **(D)** single sterols]. Bars with no letter in common are significant different (*P* < 0.05).

Tocopherols are essential natural antioxidants that are crucial in maintaining seed oil quality during processing and storage through prevention of lipid oxidation which may result in rancidity and off-flavors development (26). Figure 6A shows the effect of HA, ST, and PU pretreatments on tocopherols of camellia oil. The tocopherol content was increased by 5.28% after PU pretreatment, and decreased by 5.26% after ST pretreatment, but remained unchanged after HA pretreatment. There were many factors affecting the tocopherol content in oils. Ye et al. (13) suggested that the effects of microwave pretreatment on tocopherols of camellia oil depended the moisture contents of camellia seeds. It has also been found that the tocopherol content in oil was also related to the time of pretreatment. This is probably because tocopherols were decomposed by elevated temperature caused by relatively long period of microwave pretreatment (27). The different conditions and time of the three pretreatments resulted in different moisture content of camellia seeds, which may affect the content of tocopherols in camellia oil.

#### Squalene and sterols of camellia oil

Squalene and sterols, the major unsaponifiable components in camellia oil, have attracted increasing interest due to their health benefits, including decreasing cholesterol absorption, dementia prevention, anti-inflammatory, immunomodulatory, and anticarcinogenic activities (28). In the present study, the contents of squalene and nine sterols in camellia oil were determined to illustrate the effect of pretreatments on the active substances, and the results were shown in Figures 6B–D. The squalene content of camellia oil was decreased by 6.46, 6.91, and 4.19% after HA, ST and PU pretreatments, respectively. The difference in squalene contents in the three pretreated camellia oils was not significant.

In the present study betulin was used as an internal standard and a total of nine sterols were detected, they were Δ7-stigmasterol, sitosterol, Δ7-avenasterol, Δ5-avenasterol, stigmasta-5,24-dien-3-ol, stigmasta-5,23-dien-3-ol, clerosterol, campestrol, and 24-methylene cholesterol. The most abundant sterol in camellia oil was Δ7-stigmasterol (50%), followed by sitosterol (30%) and Δ7-avenasterol (10%). The untreated camellia oil contained 177.62 mg/100 g total sterols. All three pretreatments affected the total sterols (Figure 6C), the total sterols content in HA treated camellia oil was increased by 12.52%, while the total sterols contents in ST and PU treated camellia oil were decreased by 6.09 and 23.13%, respectively. In terms of individual sterol (Figure 6D), both HA and ST pretreatments had only minor effects on sterol content in camellia oil, with the content of Δ7-avenasterol increasing by 24.70% after HA pretreatment, and the content of 24-methylene cholesterol increasing by 37.5% after ST pretreatment, and no significant changes of the contents of other sterols in camellia oil. The PU pretreatment had the greatest effect on individual sterol of camellia oil, it increased the contents of Δ7-stigmasterol by 13.00%, stigmasta-5,24-dien-3-ol by 29.36%, and stigmasta-5,23-dien-3-ol by 64.7%, and decreased the contents of Δ7-avenasterol by 22.70%, Δ5-avenasterol by 56.82%, clerosterol by 100%, campestrol by 100%, and 24-methylene cholesterol by 100%. Similar to the microwave pretreatment, the PU pretreatment could also reduce the sterol content. According to Zhou et al. (29), microwave pretreatment (2,450 MHz, 600 W for 1, 2 and 4 min) of walnuts had a negative impact on the oil phytosterols, including total phytosterols and single phytosterol, and further increasing the microwave time to 4 min doubled the phytosterols losses.

### Effect of pretreatments on different forms of phenolics in camellia oil

**Figure 7:**
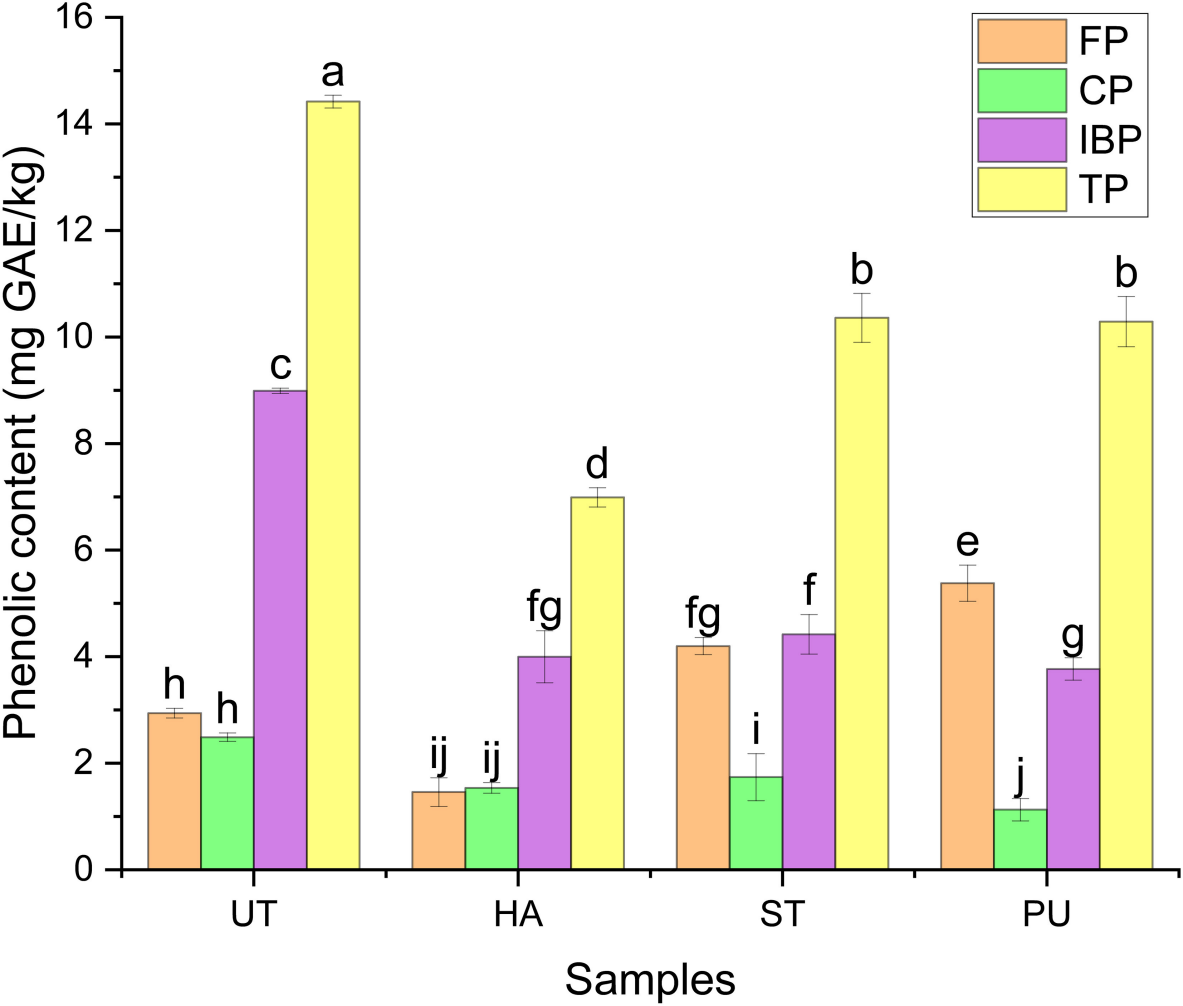
The contents of different forms of phenolics of camellia oil from untreated and HA, ST, PU pretreated camellia seeds. Bars with no letter in common are significant different (*P* < 0.05).

Phenolics are important secondary metabolites containing at least one aromatic ring and hydroxyl group, they are important bioactive functional components in plants (28). Seed oil is a good source of phenolics, it is important to improve extraction of these bioactive compounds from the seed matrix to enhance the functional properties of seed oil (26). In the present study, the contents of FP, CP and IBP in the camellia oil before and after HA, ST and PU pretreatments were determined by Folin-Ciocalteu colorimetric method, and the results are shown in Figure 7. Total phenolic (TP) content is the sum of FP, CP and IBP contents.

The most abundant phenolic form of camellia oil was IBP, which accounted for 62.34% of the TP, while FP and CP were both relatively low, they contributed about 20% of TP, respectively. Zhang et al. (30) found that the most abundant phenolic forms in camellia oil were glycosylated, esterified and insoluble, and the least abundant was free form. Luo and Fei (12) found that the average TP content of camellia species is similar both in the kernels and in the shells, and the content order of the three forms of phenolics was FP > IBP > CP, however, FP content in camellia seed oil was the lowest.

The three pretreatments had significant effects on the contents of different forms of phenolics in camellia oil. ST and PU pretreatments significantly increased the FP content of camellia oil, all three pretreatments reduced the contents of CP and IBP. A study by Gong et al. (31) showed that thermal treatment of brown rice increased the content of FP, which is similar to the findings of this study. HA pretreatment decreased the FP content of camellia oil. It is likely due to that FP are easily decomposed under high temperature (32).

### Profiles of different forms of phenolics in camellia oil

Many researchers have found different kinds of phenolics in camellia oil. Wang et al. (1) analyzed the phenolics of camellia oils from three species gathered from 15 regions of China and identified 24 phenolics. Fang et al. (33) detected a total of nine phenolic acids in camellia oil, among which 3-hydroxytyrosol, benzoic acid, catechins, 4-hydroxybenzoic acid, and chlorogenic acid were predominant. Hong et al. (34) identified 50 FP and 23 IBP in three organic camellia seed cakes.

**Figure 8:**
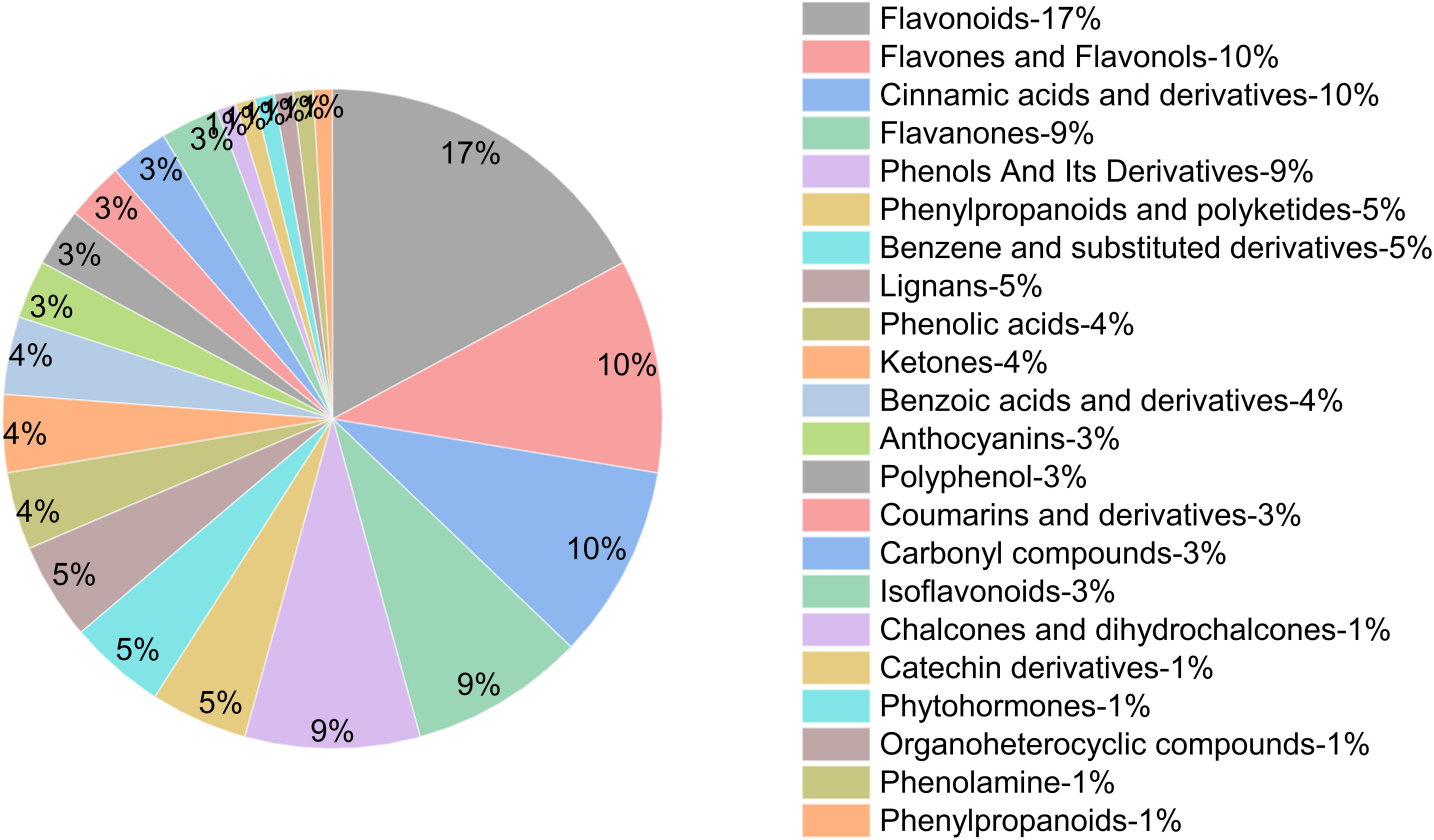
Phenolic profiles in camellia oil.

The profile of functional substances in oils are not only related to genetic characteristics, but also related to the conditions of oil processing, such as the oil preparation method and treatment temperature. To investigate the effect of pretreatments on phenolic profiles in camellia oil, three forms of phenolics were identified by non-targeted metabolomics. A total of 105 phenolics in 22 species were identified in camellia oil, with flavonoids accounting for 17%, flavones and flavonols accounting for 10%, cinnamic acid and its derivatives accounting for 9%, flavanones accounting for 8%, phenols and their derivatives accounting for 8% and other species accounting for <5% of the total (Figure 8).

In order to find the phenolics with higher significance, we screened three forms of phenolics with value importance in projection (VIP) > 1 and *P* < 0.05. Forty-three phenolics were screened for FP and CP, and 44 phenolics were screened for IBP. To investigate the effect of HA, ST and PU pretreatments on phenolics, we used fold change (FC) > 2 or FC < 0.2 as the screening threshold for differential phenolics.

**Figure 9:**
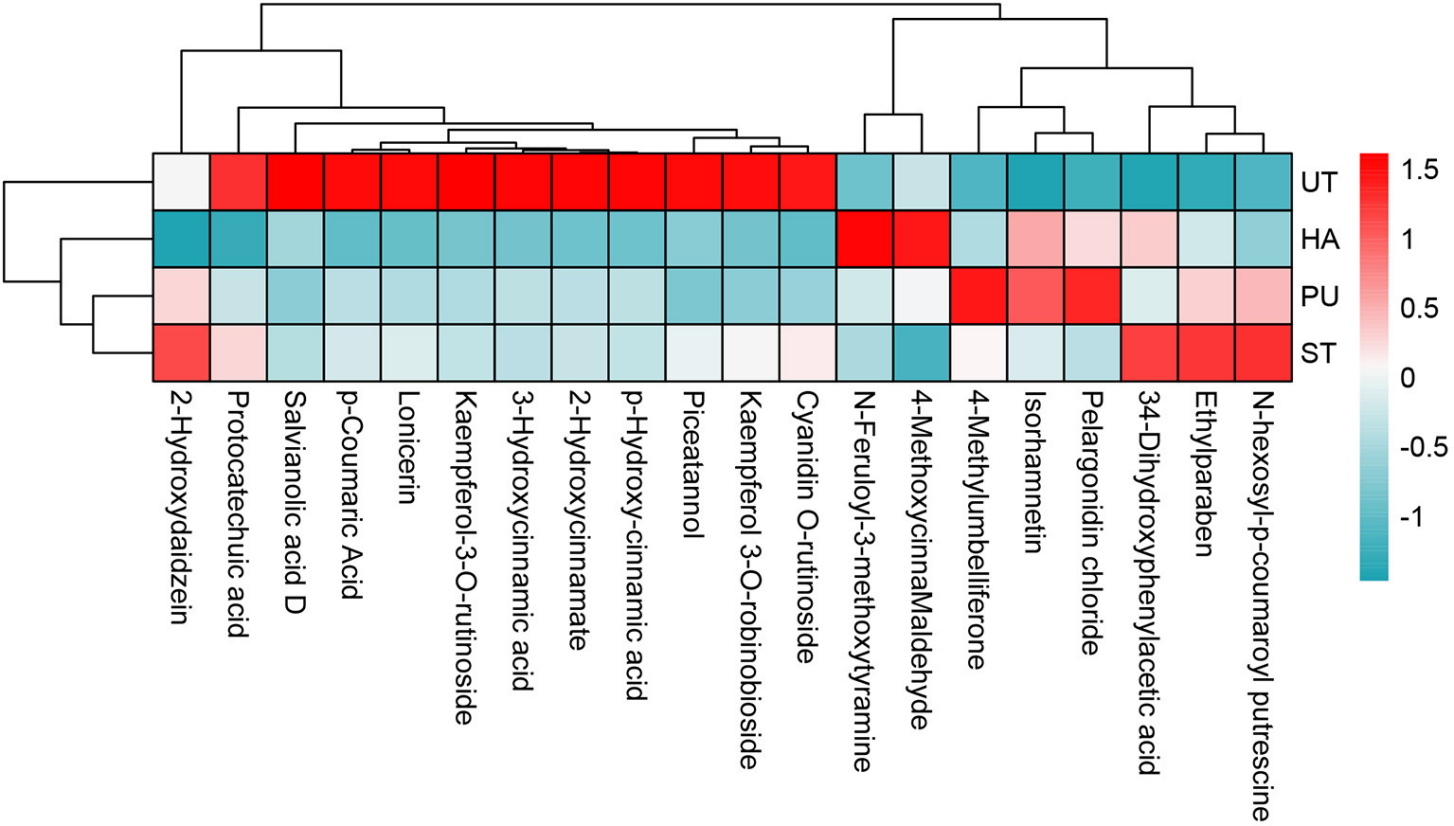
Heatmap of FP with VIP > 1 in camellia oil.

The heatmap of FP with VIP > 1 in camellia oil is shown in Figure 9. There was a total of 20 FP with VIP > 1 in camellia oil, eight of which were up-regulated and 12 of which were down-regulated. After HA pretreatment, four FP were up-regulated and 12 FP were down-regulated. Four FP were up-regulated and five FP were down-regulated after PU pretreatment, while five FP were up-regulated after ST pretreatment. In terms of individual phenolics, the contents of 4-methylumbelliferone and isorhamnetin were increased after all three pretreatments, and the content of N-feruloyl-3-methoxytyramine was increased after HA and PU pretreatments. The contents of piceatannol, kaempferol-3-O-rutinoside, kaempferol 3-O-robinobioside, cyanidin O-rutinoside, salvianolic acid D were decreased after HA and PU pretreatments.

**Figure 10:**
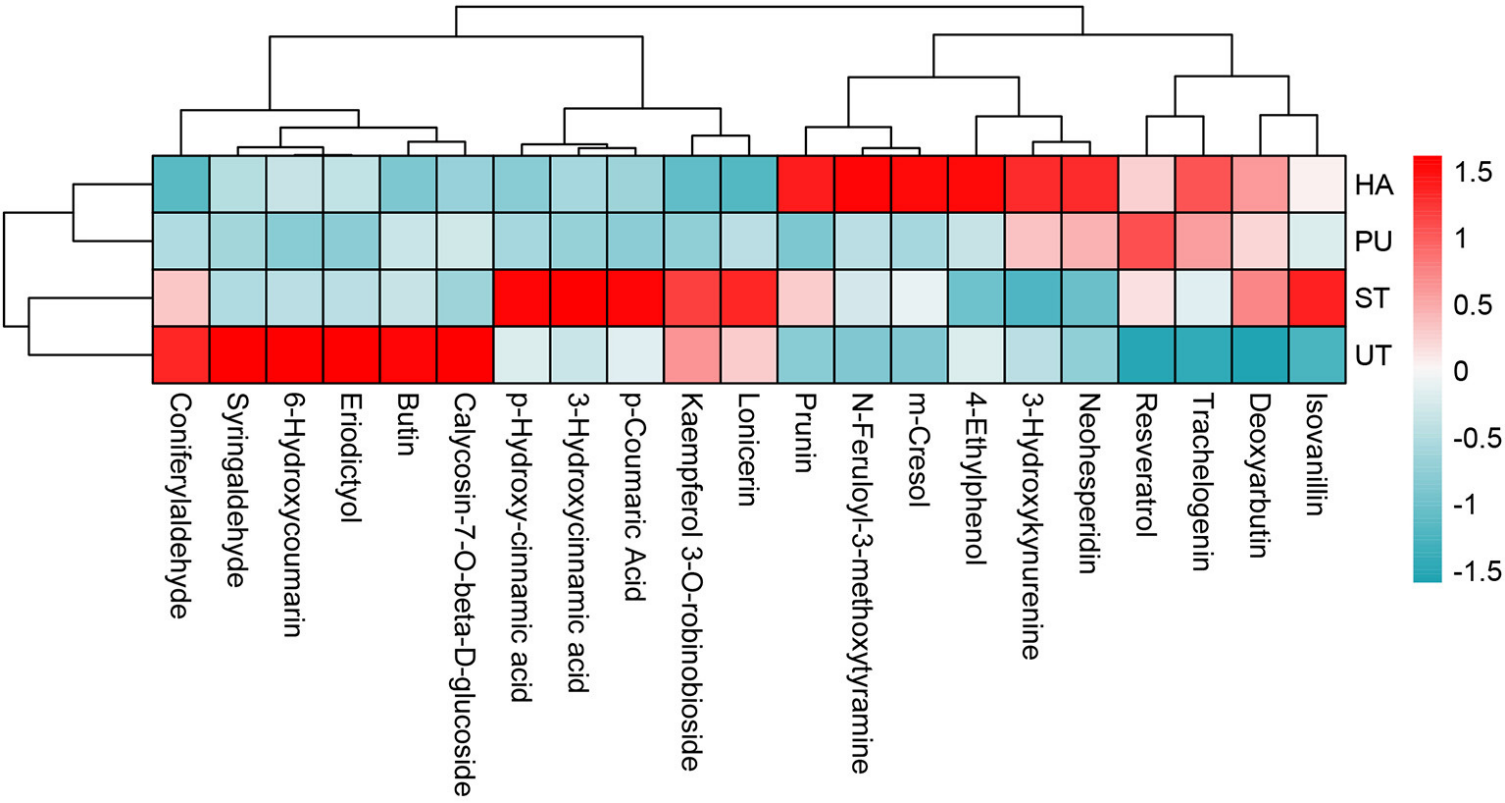
Heatmap of CP with VIP>1 in camellia oil.

The heatmap of CP with VIP > 1 in camellia oil is shown in Figure 10. There was a total of 21 CP with VIP > 1 in camellia oil, 13 of which were up-regulated and eight of which were down-regulated. Eight CP were up-regulated and six CP were down-regulated after HA pretreatment, three CP were up-regulated and three CP were down-regulated after PU pretreatment, while three CP were up-regulated and six CP were down-regulated after ST pretreatment. In terms of individual phenolics, the contents of n-feruloyl-3-methoxytyramine and trachelogenin were increased after all three pretreatments, the content of deoxyarbutin was increased after HA and ST pretreatments, the content of syringaldehyde was decreased after all three pretreatments, the content of calycosin-7-O-beta-D-glucoside was decreased after HA and ST pretreatments.

**Figure 11:**
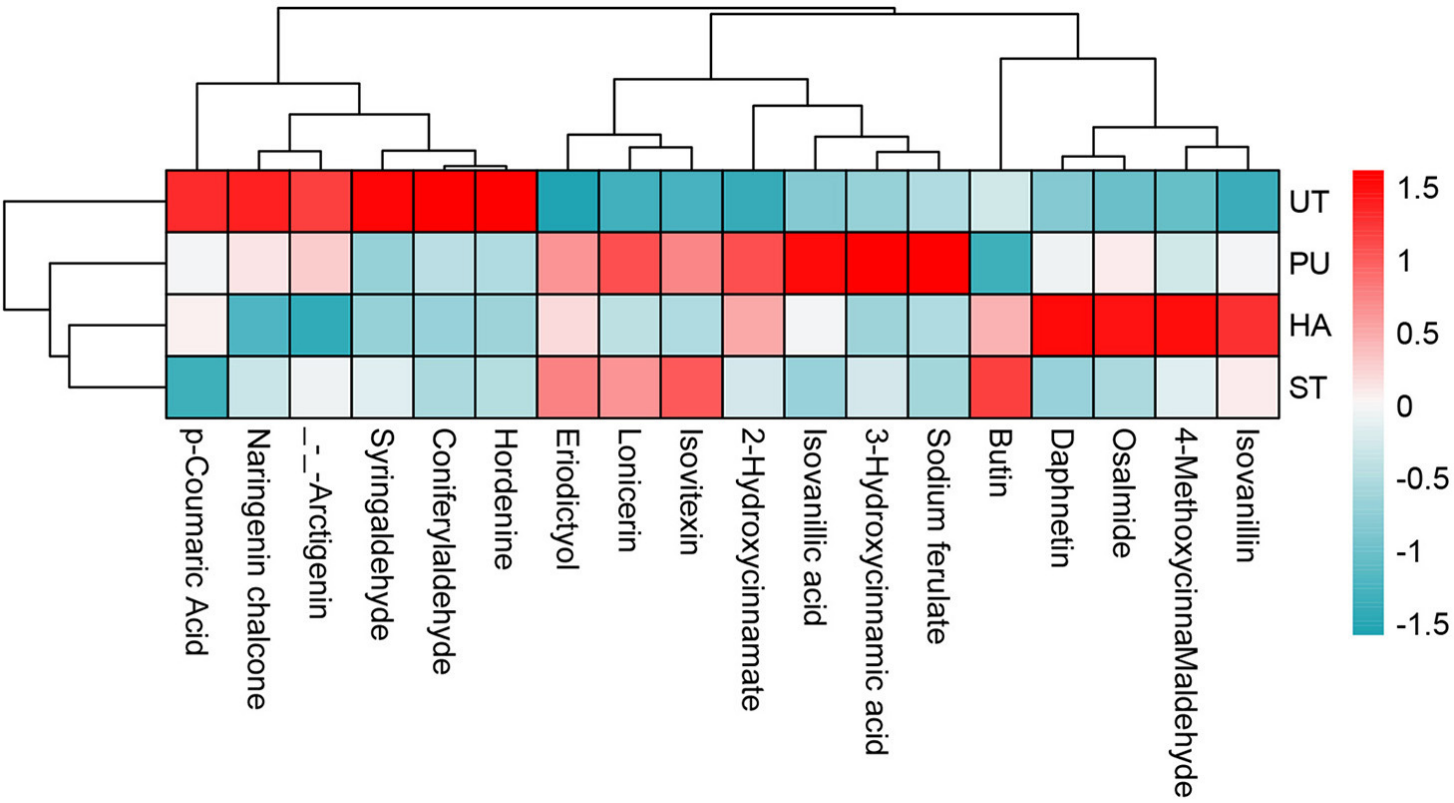
Heatmap of IBP with VIP>1 in camellia oil.

The heatmap of IBP with VIP > 1 in camellia oil is shown in Figure 11. There was a total of 18 IBP with VIP > 1 in camellia oil, 12 of which were up-regulated and six of which were down-regulated. Six IBP were up-regulated and six IBP were down-regulated after HA pretreatment, eight IBP were up-regulated and three IBP were down-regulated after PU pretreatment, while three IBP were up-regulated and two IBP were down-regulated after ST pretreatment. In terms of individual phenolics, the content of eriodictyol was increased after all three pretreatments, the contents of osalmide and 2-hydroxycinnamate were increased after HA and PU pretreatments, and the content of lonicerin was increased after PU and ST pretreatments, while the contents of coniferylaldehyde and hordenine were decreased after all three pretreatments, and the content of syringaldehyde was decreased after HA and PU pretreatments.

### Antioxidant capacities of FP, CP, and IBP in camellia oil

**Figure 12:**
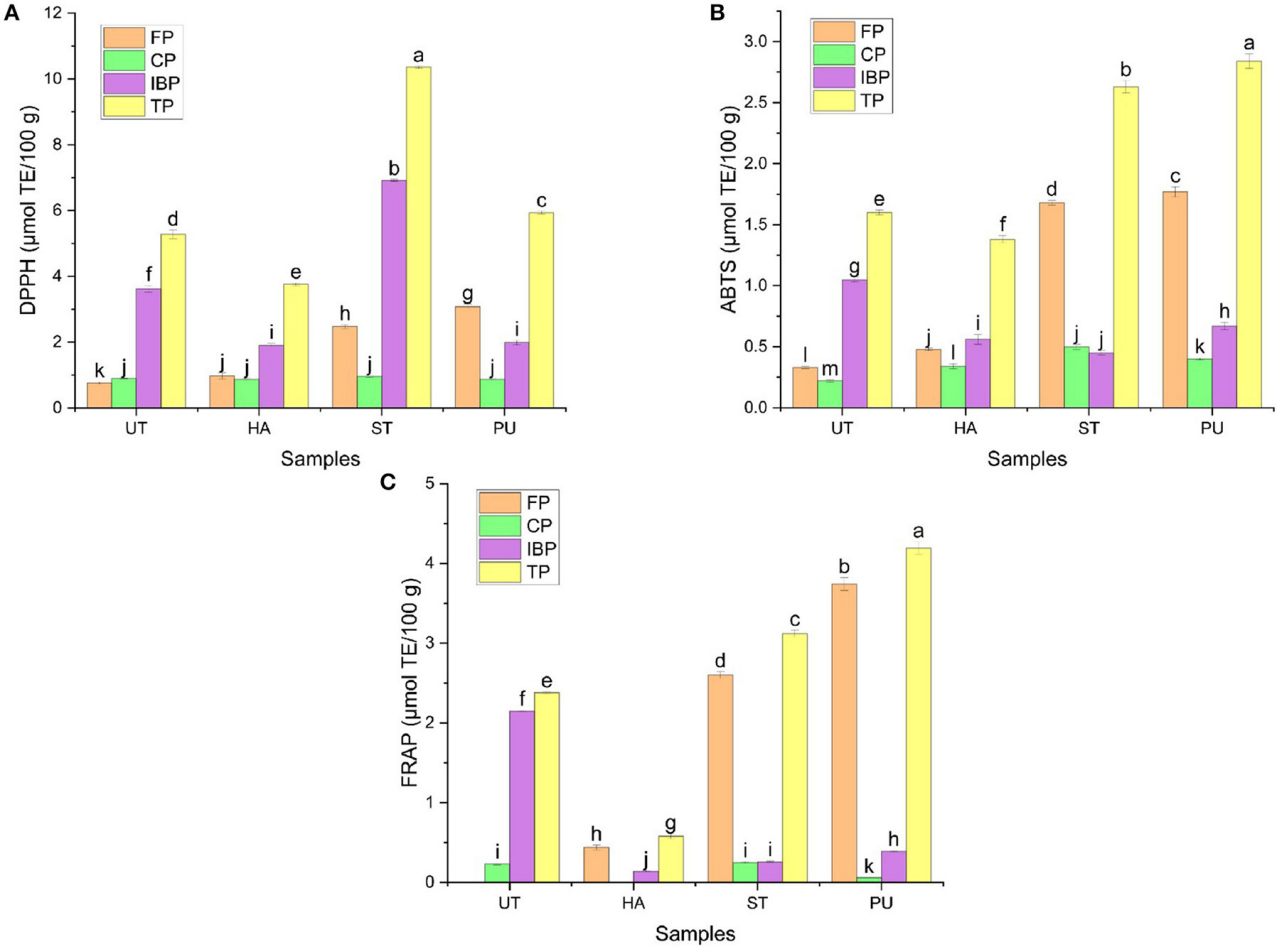
The antioxidant capacities of FP, CP, and IBP of camellia oil from untreated and HA, ST, PU pretreated camellia seeds. [**(A)** DPPH, **(B)** ABTS, **(C)** FRAP]. Bars with no letter in common are significant different (*P* < 0.05).

The antioxidant properties of phenolics in oils have been reported for a long time (10), but the antioxidant capacities of different forms of phenolics showed significant variations (35). To better evaluate the antioxidant capacities of phenolics in camellia oil, three antioxidant methods, i.e., DPPH, ABTS radical scavenging methods, and FRAP were used in the present study, and the results are shown in Figure 12. The DPPH, ABTS and FRAP of IBP in camellia oil were higher than those of FP and CP. Similar to the results reported by Wang et al. (35), IBP was major contributor to the antioxidant capacity of camellia oil. Moreover, IBP in food are more stable than soluble free antioxidants, so a large proportion of IBP can reach the colon before decomposition and exert their antioxidant effects (36).

The DPPH, ABTS scavenging capacities and FRAP of FP in camellia oil were significantly increased after pretreatments. HA and PU pretreatments significantly reduced the DPPH scavenging capacities of IBP, but ST pretreatment significantly increased the DPPH scavenging capacity of IBP by 91.16%. In general, ST and PU pretreatments increased DPPH, ABTS and FRAP of total phenolics in camellia oil, but HA decreased DPPH, ABTS and FRAP of total phenolics in camellia oil, indicating that ST and PU pretreatments had a positive effect on the antioxidant capacities of phenolics.

The correlation between phenolics in camellia oil and antioxidant capacity was analyzed, and the results are presented in Table 1. IBP was significantly correlated with FRAP (*r* = 0.969, *P* < 0.01). A total of eight phenolics showed correlations with antioxidant activity. In FP, 4-methylumbelliferone was significantly correlated with FRAP (*r* = 0.951, *P* < 0.05). In CP, 3-hydroxycinnamic acid (*r* = 0.977, *P* < 0.05), *p*-coumaric acid (*r* = 0.993, *P* < 0.01), *p*-hydroxycinnamic acid (*r* = 0.989, *P* < 0.05) were significantly correlated with DPPH, and kaempferol-3-O-robinobioside were significantly correlated with FRAP (*r* = 0.989, *P* < 0.05). As for IBP, coniferylaldehyde (*r* = 1.000, *P* < 0.01), hordenine (*r* = 0.997, *P* < 0.01), syringaldehyde (*r* = 0.966, *P* < 0.05) were significantly correlated with FRAP. The antioxidant activity of edible oils has been ascribed to the presence of phenolics. Phenolics in edible oils possess several health benefits such as antioxidant, antibacterial, antiviral, anti-inflammatory, anti-tumor, cardioprotective, neuroprotective, anti-diabetic, and anti-obesity activities (37–39).

**Table 1:** Correlation of phenolics in camellia oil with antioxidant capacity.

**Phenolics**	**FP**					**CP**								**IBP**						
	**FP**	**4-** **Methylumbelliferone**	**DPPH**	**ABTS**	**FRAP**	**CP**	**3-Hydroxycinnamic acid**	**p-Coumaric acid**	**p-Hydroxycinnamic acid**	**Kaempferol** **-3-O-** **robinobioside**	**DPPH**	**ABTS**	**FRAP**	**IBP**	**Coniferyl-** **aldehyde**	**Hordenine**	**Syring-** **aldehyde**	**DPPH**	**ABTS**	**FRAP**
DPPH	0.850	0.933	1			−0.534	0.977*	0.993**	0.989*	0.908	1			0.138	0.013	0.049	0.237	1		
ABTS	0.834	0.866	0.986*	1		−0.761	0.674	0.596	0.634	0.174	0.524	1		0.816	0.947	0.931	0.843	−0.293	1	
FRAP	0.812	0.951*	0.999**	0.976*	1	−0.269	0.703	0.767	0.782	0.989*	0.837	0.057	1	0.969**	1.000**	0.997**	0.966*	0.002	0.953*	1

*Significant correlation at 0.05 level (two-tailed).

**Significant correlation at 0.01 level (two-tailed).

## Conclusion

In the present study, three different pretreatments (HA, ST, and PU) were used to pretreat the camellia seed powder before camellia oil production. The effect of pretreatments on camellia seeds and oil was investigated. Pretreatments of camellia seed significantly affected the oil yield of camellia seeds and the quality, antioxidant properties of camellia oil. Particularly, PU pretreatment showed some advantages as it not only improved the oil yield of camellia seeds, but also increased the contents of tocopherols and phenolics of camellia oil, and enhanced the antioxidant capacity of phenolics in camellia oil. This paper could provide some theoretical guidance for the pretreatment of camellia oil or other vegetable oil production.

## Data availability statement

The raw data supporting the conclusions of this article will be made available by the authors, without undue reservation.

## Author contributions

MW: conceptualization, methodology, validation, data curation, writing—original draft preparation, visualization, and funding acquisition. YZ: software and validation. YW: resources, writing—review and editing, and funding acquisition. QZ: formal analysis and validation. LS: validation and software. EG: writing—review and editing, funding acquisition, and supervision. GF: conceptualization and resources. All authors contributed to the article and approved the submitted version.

## Funding

This work was supported by the National Natural Science Foundation of China (31360391), Science and Provincial key R&D Program of Jiangxi (20192BBF60037), and High-level Talents' Scientific Research Foundation of Gannan Medical University (QD202123).

## Conflict of interest

The authors declare that the research was conducted in the absence of any commercial or financial relationships that could be construed as a potential conflict of interest.

## Publisher's note

All claims expressed in this article are solely those of the authors and do not necessarily represent those of their affiliated organizations, or those of the publisher, the editors and the reviewers. Any product that may be evaluated in this article, or claim that may be made by its manufacturer, is not guaranteed or endorsed by the publisher.
